# The MicroRNAs in the Pathophysiology of Osteoporosis

**DOI:** 10.3390/ijms25116240

**Published:** 2024-06-05

**Authors:** Julia Trojniak, Anna Sendera, Agnieszka Banaś-Ząbczyk, Marta Kopańska

**Affiliations:** 1 Student Research Club “Reh-Tech”, Medical College of Rzeszow University, 35-959 Rzeszow, Poland; juliatrojniak0@gmail.com; 2 Department of Biology, Institute of Medical Sciences, Medical College of Rzeszow University, 35-959 Rzeszow, Poland; antrzyna@ur.edu.pl (A.S.); agnieszkabanas@o2.pl (A.B.-Z.); 3 Department of Pathophysiology, Institute of Medical Sciences, Medical College of Rzeszow University, 35-959 Rzeszow, Poland

**Keywords:** microRNA, osteoporosis, miRNA, mRNA

## Abstract

Globally, osteoporosis is the most common systemic skeletal disease. There are many factors that influence osteoporosis’ development and progression. During the pathogenesis of this disease, bone turnover is imbalanced between resorption and the formation of bone tissue. A growing interest has been devoted to the role that microRNA (miRNA) plays in osteoporosis regulation. A microRNA (miRNA) is a group of small single-stranded RNA molecules involved in regulating gene expression in eukaryotic organisms. As microRNAs (miRNAs) are key regulators of gene expression and can modulate processes related to bone metabolism, they have become increasingly important for studying osteoporosis pathogenesis. The available research suggests that miRNAs play an important role in regulating processes associated with bone metabolism, especially by influencing bone resorption and synthesis. Furthermore, microRNAs can also serve as potential therapeutic targets for osteoporosis, besides being a rapid and specific biomarker.

## 1. Introduction

Osteoporosis is a chronic skeletal disease that increases the risk of bone fractures due to decreased mechanical strength [1]. A bone’s mechanical resistance is determined by its bone mineral density (BMD) and bone tissue quality [2]. Over the course of a person’s life, the bone undergoes constant reconstruction, which is governed by osteoblasts, which create tissue, osteoclasts, which resorb it, and osteoocytes, which control its structure [3]. Basically, osteoporosis is caused by an imbalance between osteoblasts and osteoclasts, leading to excessive bone resorption in the absence of bone formation. The remodeling of bone tissue is closely related to changes in calcium and phosphate metabolism. There are local and systemic factors that influence bone tissue as well. The majority of these factors interfere with osteoblast differentiation and osteoclast activity, resulting in osteoporosis development [2].

As osteoporosis can present asymptomatically and its first sign is typically a fracture, the condition is often detected late in its progression [4]. This increases the risk of disabilities and impaired functioning for the patient [5]. The development of better treatments for osteoporosis is not the only way to prevent bone loss, but it is also important to identify patients at high risk of developing osteoporosis, especially when the number of osteoporosis patients is expected to rise, as it is in aging societies [6]. The development of new diagnostic biomarkers for osteoporosis based on early molecular events is therefore urgently needed. Osteoporosis molecular biomarkers may also contribute to the development of alternative therapeutic strategies as well as to the development of clinical indicators of response to interventions involving the administration of drugs that prevent or modify the disease.

In recent years, it has been demonstrated that some RNAs, such as microRNAs and long noncoding RNAs (lncRNAs), regulate gene expression and impact many biological processes, including bone metabolism [7].

**Figure 1:**
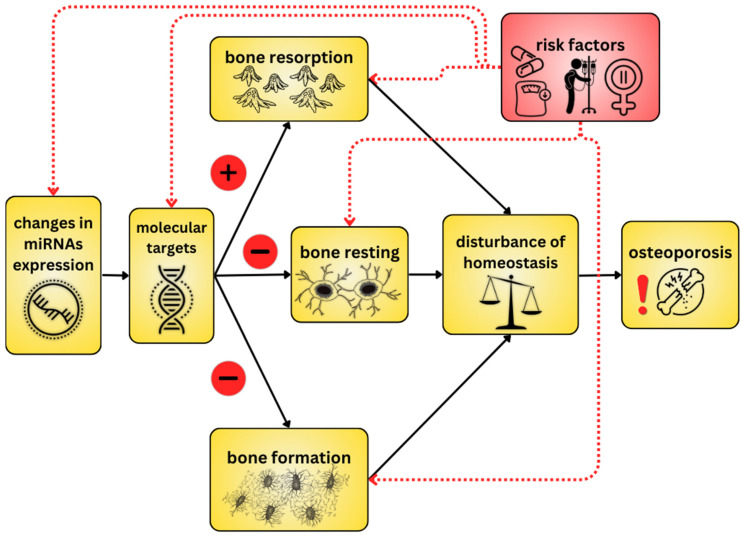
An overview of osteoporosis development including miRNAs. As a result of changes in miRNAs, molecular targets (target genes and signaling pathways) are disrupted, leading to bone homeostasis disruption through several pathways—such as increased bone resorption, decreased bone formation, and disruption of resting bone. Individual components may be affected by risk factors.

The microRNAs, or miRs, are tiny, single-stranded, and non-coding molecules derived from double-stranded precursors [8,9,10]. As post-transcriptional regulators, they affect messenger RNA (mRNA) translation or stability and thus regulate gene expression. Gene expression is regulated by base complementarity between the seed region of the miRNA and the 3′ untranslated region (UTR) of the target mRNA [1]. It is estimated that hundreds of microRNA genes are present in the genomes of plants, animals, and humans [8,11]. Biological processes that miRNAs influence include proliferation, differentiation, angiogenesis, migration, and apoptosis [12]. Evidence suggests that miRNAs are involved in the pathogenesis of numerous diseases, such as diabetes and cancer [12,13,14,15]. In addition, recent reports suggest that miRNAs are important for regulating bone formation and homeostasis in the pathogenesis of osteoporosis [16]—Figure 1. In addition, miRNAs are present in body fluids, including serum, suggesting that miRNAs could serve as simple biomarkers for osteoporosis [17,18,19,20,21,22,23].

## 2. Bone Remodeling Process

As a result of a disturbance in the bone remodeling process, osteoporosis may develop. The skeletal system contains a variety of cell types that work together to maintain bone homeostasis [24]. The processes that determine homeostasis are bone formation (ossification) and bone resorption [25]. As a result of them, the bones of humans undergo dynamic changes throughout their lives. In a normal physiological state, bone formation and bone resorption are balanced [24]. The key molecular mediator of bone remodeling is a system consisting of three components: RANK, RANK ligand (RANKL), and osteoprotegerin (OPG) [25].

Bone formation is controlled by bone cells of mesodermal origin, which are precursors of osteoblasts, including bone marrow stem cells (BMSCs) [24,25]. Osteoblasts are responsible for the deposition of minerals and the formation of a collagenous bone matrix. They are supported by multifaceted osteoocytes, the most numerous cell type with a wide network of tubules [24]. In turn, osteoclasts, multinucleated cells derived from hematopoietic stem cells, are responsible for the resorption of bone matrix. They are regulated by extracellular signals secreted by osteoblasts and osteoocytes. This process is supported by precursors such as bone marrow monocytes or macrophage precursors. Osteoclast differentiation requires the binding of RANK receptors on osteoclast surfaces to the RANKL ligand secreted by osteoblasts and bone marrow cells. This contributes to resorption activation [24]. In addition, substances that influence osteoclast differentiation include parathyroid hormone (PTH) and calcitonin, cholecalciferol (vitamin D3), interleukin 1 (IL-1), interleukin 6 (IL-6), interleukin 11 (IL-11), TNF- and osteoprotegerin (OPG protein). OPG acts as a receptor that blocks RANKL binding to RANK receptors, which regulate osteoclast activity. With age, the number of osteoprogenitors and osteoblasts declines, leading to reduced OPG levels and osteoclast activity predominance, which can contribute to osteopenia and osteoporosis [24]. Bone matrix resorption leads to the release of proteins such as TGF-β1 and IGF-1, which promote the differentiation of mesenchymal cells into osteoblasts. Osteocytes, degraded osteoblasts, can be a source of RANKL, which stimulates osteoclastogenesis. Osteocyte-specific RANKL deletion may cause osteopetrosis. Estrogen deficiency and inflammation are also known to cause bone resorption, primarily through increased production of inflammatory cytokines such as interleukin 1 (IL-1), interleukin 17 (IL-17), tumor necrosis factor-α (TNF-α), and receptor activator nuclear factor -κB (RANKL) in the bone marrow, which induce an increase in osteoclast production, activation, and survival [1]. Increased osteoclastogenesis is responsible for osteolysis, which is a serious consequence of inflammation-related diseases associated with bone destruction. The mechanisms that limit osteoclastogenesis in inflammatory conditions are largely unknown [26]. Bone remodeling is divided into five basic phases.

### 2.1. Activation Phase

Bone remodeling is triggered by a local mechanical or hormonal stimulus. Upon capturing a signal, osteoocytes transmit it to the bone to initiate a biological response [27]. On the surface of the bone, osteoclasts are recruited [28]. As a result of the activation phase, local factors such as TGF-β, macrophage colony-stimulating factor (M-CSF), receptor activator of NF-κB ligand (RANKL), and systemic regulators, i.e., vitamin D, calcium, PTH, estrogen, androgen, and glucocorticoid will promote osteoclastogenesis and a new round of remodeling will begin.

### 2.2. Resorption Phase

Mature osteoclasts secrete matrix metalloproteinases (MMPs), generating an acidic microenvironment between the cell and the bone surface to digest both the mineral and organic matrix. In this phase, resorption lacunae are formed under the canopy cells Howship [27,28,29].

### 2.3. Reversal Phase

Apoptosis is induced in osteoclasts and osteoblasts are recruited to the surface of the bone. During this phase, molecules such as TGF-β are released locally and attract osteoblasts to initiate bone formation [28,30].

### 2.4. Formation Phase

This process usually takes 4–6 months [31]. There are a number of local and systemic hormones that promote osteoblastogenesis in bone during this stage, including Wnt, sclerostin, and parathyroid hormone (PTH) [30]. An organic bone matrix (osteoid) composed of various proteins such as type I collagen begins to build up until bone resorption is fully compensated. they deposit collagen. It is mineralized to form new bone [28].

### 2.5. Completion Phase

The amount of bone matrix is resorbed and created; the formation phase is completed. In this phase, osteoblasts will either undergo apoptosis or form new osteocytes. Bone mineralization will begin and end in this phase [28,30,31].

A better understanding of the processes and factors that influence bone repair and homeostasis is essential to developing new diagnostic and treatment strategies.

## 3. Osteoporosis Risk Factors

In order to understand how osteoporosis develops and progresses, as well as how the disease is treated, numerous factors need to be taken into consideration. Identifying potential osteoporosis risk factors is crucial to choosing the right treatment.

There are two types of osteoporosis: primary osteoporosis and secondary osteoporosis. Primary osteoporosis categories include senile osteoporosis, postmenopausal osteoporosis, and adolescent osteoporosis, while secondary osteoporosis is caused by diseases and drugs [32]. As our society ages, primary osteoporosis prevalence increases much more than secondary osteoporosis [33].

The mineral density and quality of bones are altered in osteoporosis as a result of a variety of factors, including changes in reproductive status, genetic factors, lifestyle, nutrition, diseases, and medication use—Table 1.

## 4. The Role of miRNAs in the Pathogenesis of Osteoporosis

The mode of action of miRNAs is to recognize the 3′-untranslated region (3′-UTR) of target mRNAs through sequence complementarity and recruitment of nucleases to inhibit mRNA expression [37,38,39]. In miRNAs, the seed region contains two to eight nucleotides that are conserved across the family and direct target recognition. Complementarity between target mRNAs and miRNAs determines their fate [37]. High complementarity results in mRNA degradation, while partial complementarity prevents the translation of target mRNAs [40].

It is known that miRNAs play a key role in osteoporosis—Table 2. Different miRNAs have different properties and modulate bone metabolism using various targets. It is also worth mentioning that the impact on osteoporosis development and course associated with miRNA may be regulated by the intestinal microbiota. Specific gut bacteria can influence immune responses in the body, which has profound implications for bone health and other aspects of osteoporosis [1].

MicroRNAs may be an effective, convenient, and rapid diagnostic marker for osteoporosis [18,22]. Following these studies, osteoporosis-related miRs and the genes and proteins they modulate are receiving increasing attention. It has become increasingly common to discuss this topic in recent years.

### 4.1. miRNAs Related to Osteoblast Differentiation and Proliferation

**Figure 2:**
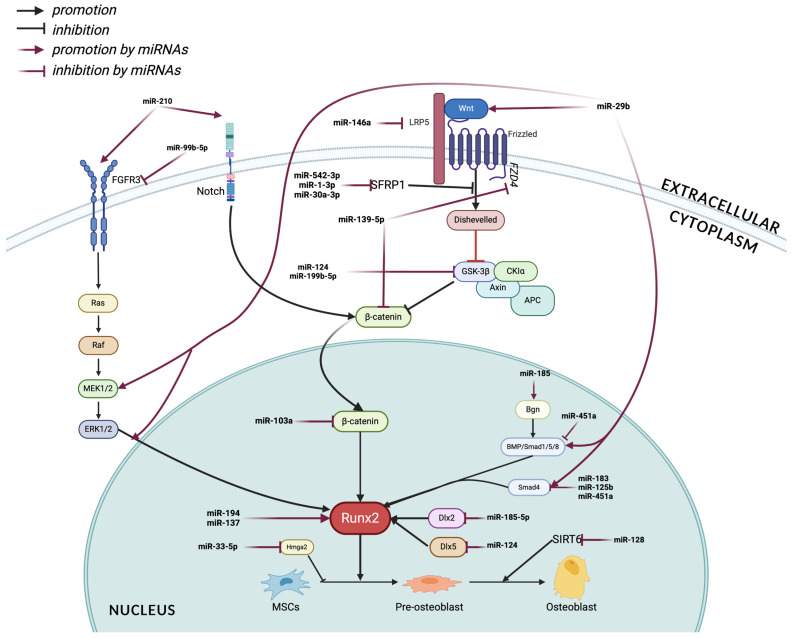
Schematic diagram of the molecular mechanisms involved in osteoblast-associated miRNAs that contribute to osteoporosis pathophysiology. Created with BioRender.com.

The microRNAs (miRNAs) are important regulators of gene expression that influence various biological and pathophysiological processes, such as bone metabolism. Maintaining bone homeostasis is dependent on osteoblasts, which produce bone matrix and mineralize it. More and more research has been conducted in recent years to understand how miRNAs modulate osteoblast function and their role in osteoporosis pathogenesis. The identification of osteoblast-related miRNAs that influence osteoporosis development may provide new therapeutic options and biomarkers for early diagnosis and monitoring. A number of miRNAs play a role in osteoblast function and osteoporosis pathogenesis by regulating specific molecular targets—Figure 2.

#### 4.1.1. miR-185

According to Yau et al., miR-185 downregulation increased osteoblast viability and reduced cell apoptosis, while miR-185 mimics inhibited cell growth and proliferation by lowering the expression of β-catenin and Wnt5b [76]. Also, miR-185 modulates tumor growth factor (TGF-1) expression after ankle fracture within two weeks, indicating it might play a role in fracture regeneration. MiR-185 is also capable of modulating osteoblast differentiation and mineralization, and mice with miR-185 deficiency showed reduced bone loss during osteoporosis [41]. Another study found that MiR-185-5p was upregulated in Runx2 mutant cells and inhibited osteogenesis in MC3T3-E16 cells [77].

The miR-185-5p family has been shown to regulate amelogenesis and osteoblast differentiation and may affect osteogenesis as well. MiR-185-5p was shown to act as a negative regulator of osteoblast differentiation by repressing the transcription factor Dlx2. The miR-185-5p-Dlx2 axis appears to contribute to abnormal bone and tooth development. However, it is not clear whether miR-185-5p is expressed in cartilage, bone, or both [51]. It nevertheless sheds new light on this molecule’s potential as an osteoporosis marker.

#### 4.1.2. miR-124

Over the past few years, scientists have intensively analyzed the links between miR-124 and the process of bone resorption. As an example, research conducted by Lee’s research group showed that miR-124 acts as an osteoclast-genesis inhibitory factor by affecting the NFATc transcription factor [43]. It has been demonstrated that miR-124 inhibits osteoblast differentiation by targeting the transcription factors Dlx5, Dlx3, and Dlx2 and accelerates osteogenic differentiation and bone formation in vivo [42]. Downregulation of miR-124 expression increased GSK-3β expression, attenuated the activity of the Wnt/β-catenin pathway, and inhibited the differentiation of ligament fibroblasts into osteoblasts. These results indicate that miR-124 contributes to the inhibition of osteoblast differentiation by inhibiting GSK-3β expression, activating the Wnt/β-catenin pathway, and promoting the differentiation of ligament fibroblasts into osteoblasts [44].

#### 4.1.3. miR-33-5p

The miR-33-5p gene is capable of sensing multiple mechanical environments in osteoblasts, thereby modulating osteoblast differentiation in response to both contact and noncontact forces. Specifically, miR-33-5p promotes osteoblast differentiation by directly targeting the 3′ UTR of its immediate target, Hmga2 [45]. MiR-33-5p’s role in cancer has also been proven [78].

#### 4.1.4. miR-103a

MiR-103a, in turn, directly targets a key transcription factor associated with osteoblast differentiation and, at the same time, the main regulator of osteogenesis—Runx2. The demonstrated mechanism of action was interaction with its 3′ UTR during cyclic mechanical stretch (CMS)-induced osteoblast differentiation [46]. Based on Zuo et al.’s study, miR-103a negatively correlates with CMS-induced osteogenesis. Additionally, miR-103a inhibited osteoblast activity and matrix mineralization in vitro [46].

#### 4.1.5. miR-139-5p

Using human bone marrow mesenchymal stem cells (hBMSCs), Long et al. investigated miR-139-5p’s role in osteogenic differentiation. By inhibiting miR-139-5p, osteoblast differentiation of hBMSCs was significantly enhanced, whereas miR-139-5p overexpression limited it. These results were derived from analysis of changes in alkaline phosphatase (ALP) activity, alizarin red staining (ARS), and the expression of genes related to osteogenesis, such as runt-2 (Runx2), collagen I (Col-1), and osteocalcin (OCN). The role of miR-139-5p in hBMSC osteogenesis is probably due to the regulation of the Wnt/β-catenin pathway through direct effects on CTNNB1 and frizzled 4 (FZD4), key components of this pathway [47].

#### 4.1.6. miR-194

MiR-194 targeted a similar molecular target and overexpression of miR-194 significantly increased osteoblast differentiation. Data indicated that STAT1, an important signaling molecule in interferon signaling pathways, was a target gene of miR-194, through which miR-194 could also regulate Runx2 activity and osteoblast differentiation in BMSCs [79]. Interestingly, STAT1-deficient mice were shown to exhibit increased fracture remodeling and membrane ossification in a mouse model of bone fractures [48]. There are also previous reports that suggest that interferon systems, including interferon-α, β, and γ, are involved in the regulation of interferon systems by inhibiting osteoclastogenesis, in which STAT1 is necessary to terminate interferon-mediated signaling [48]. In vitro data showed that STAT1 negatively regulates osteoblast differentiation by repressing Osx transcription through inhibition of p65 activity [79].

#### 4.1.7. miR-199b-5p

Zhao et al. examined the relationship between miR-199 expression and bone metabolism in osteoporosis patients. According to the researchers, there was a significant reduction in the level of miR-199b in the plasma of osteoporotic patients compared to controllers. MiR-199b-5p is induced during osteoblast differentiation in BMSCs and acts as an activator of osteoblast differentiation because knockdown of miR-199b-5p reduces, while its overexpression enhances, the differentiation process. MiR-199b-5p is associated with alkaline phosphatase (ALP) and Runx2 expression, as well as ALP activity. Moreover, miR-199b-5p regulates osteoblast differentiation of BMSCs through the GSK-3β/β-catenin signaling pathway during osteogenesis. Collectively, miR-199b-5p acts as a positive regulator in osteoblast differentiation of BMSCs [49]. In another study, miR-199a-5p increased osteogenic differentiation in vivo and in vitro by targeting the interferon-induced protein Tetratricopeptide Repeats 2 (IFIT2) [50]. Data suggest that miR-199b may be important for bone homeostasis and may represent a potential biomarker for osteoporosis [49,50].

#### 4.1.8. miR-542-3p

Researchers Zhang et al. used rat MSCs and ovariectomized rats (OVX) for their study. Using rat MSCs, their study demonstrated that miR-542-3p affects osteoblast differentiation in vitro. A miR-542-3p-mediated increase in osteoblast-specific markers was observed. Additionally, they investigated the effect of miR-542-3p on rats with osteoporosis induced by ovariectomy. Inhibition of miR-542-3p promoted SFRP1 protein expression, reduced bone mass and bone formation, and worsened bone loss in OVX rats. While miR-542-3p mutant expression did not inhibit SFRP1 protein expression, bone mass, or bone formation in OVX rats [52].

#### 4.1.9. miR-146a

Liu et al. found that miR-146a plays an important role in osteoporosis pathophysiology and that inhibiting it has beneficial effects. The researchers studied how miR-146a affected OP in the jaws of OVX rats in their experiment. MiR-146a downregulation inhibited OP in rats with removed ovaries by activating the Wnt/β-catenin signaling pathway [53].

#### 4.1.10. miR-128

According to Zhao et al., MiR-128 inhibits osteogenic differentiation in osteoporosis by downregulating SIRT6 expression. In addition, miR-128 overexpression significantly inhibited the expression of osteocalcin (OC), alkaline phosphatase (ALP), and collagen type I-α1 (COL1A1) in C2C12 cells, while miR-128 inhibitors reversed this effect [54].

#### 4.1.11. miR-183

In the study by Qin et al., the expression of miR-183 in osteoporosis was determined using ovarian-bearing (OVX) mice. The results showed that miR-183 was upregulated in osteoporosis, and miR-183 overexpression inhibited osteoblast differentiation by deliberately downregulating the TGF-β–Smad4 pathway member to induce osteoporosis [55].

#### 4.1.12. miR-99b-5p

An analysis of online datasets by Ding et al. revealed that miR-99b-5p was abnormally upregulated in osteoporosis and downregulated in differentiated osteoblasts. It has been found that miR-99b-5p is upregulated in osteoporosis bone tissues, and FGFR3 is downregulated. Overexpression of miR-99b-5p inhibited osteoblast proliferation and osteogenesis-related gene expression, whereas overexpression of FGFR3 had the opposite effects. FGFR3 expression was inhibited by miR-99b-5p by directly targeting it [56].

#### 4.1.13. miR-137

Cai et al. found that osteoporotic rats with hormone-induced osteoporosis had higher miR-137 expression levels in their bone tissues. Rats with osteoporosis whose miR-137 has been silenced have higher bone mineral density and an increase in BGP and TALP levels. According to the above results, miR-137 is closely related to osteoporosis and bone mineral density decreases in osteoporotic model rats. It has been suggested that miR-137 targets RUNX2. It was found that mice with hormone-induced osteoporosis exhibited high expression of miR-137, which decreased the expression of RUNX2 in bone tissues, while mice with knockdown of miR-137 exhibited increased expression of RUNX2 [57].

#### 4.1.14. miR-29

It is thought that miR-29 is involved in bone remodeling, including collagen synthesis, which may hold significance for osteoporosis. Many scientific papers have been published on this topic. For example, a study by Zhang et al. suggests that miR-29 may be a key factor in the pathogenesis of osteoporotic bone lesions [80]. Researchers showed that miR-29a/b/c was markedly reduced in the bone tissue of osteoporotic patients compared to controls. Moreover, an inverse correlation was found between the level of miR-29 expression and osteoporotic changes in bone mineral density. The results suggest that miR-29 may regulate collagen synthesis, which is imperative for maintaining bone integrity. Recent reports also indicate that miR-29b is significantly reduced in the bone tissue of osteoporotic patients compared to controls. Additionally, miR-29b has been shown to regulate the expression of genes related to the synthesis of type I collagen, which may affect bone structure and strength [81]. Overexpression of miR-29b was implicated in osteoblast differentiation in the MC3T3-E1 cell line [58]. Hence, miR-29b modulates bone extracellular matrix proteins and anti-osteogenic factors to regulate osteoblast phenotypes [58]. This study suggests that miR-29 has an important role to play in the regulation of osteoporosis, possibly by influencing collagen synthesis and bone tissue integrity through the ability to regulate processes related to osteoporosis.

#### 4.1.15. miR-451a

MiR-451a is essential for osteogenesis, which has been demonstrated in in vitro and in vivo experiments. However, studies in osteoporotic mice showed that MiR-451a negatively modulates Bmp6 and Bmp6R expression during osteoblastogenesis. A reduction in miR-451a led to an increase in bone mass, likely due to phosphorylation of SMAD1/5/8 signaling. Moreover, mice overexpressed with miR-451a resulted in lower levels of collagen I as compared with controls [16]. According to these findings, miR-451a could modulate osteoblast differentiation and mineralization, and suppressing this factor may be a useful therapeutic strategy for treating osteoporosis.

#### 4.1.16. miR-30a-3p

A study by Liu et al. investigated the effect of miR-30a-3p on osteoporosis in ovariectomized rats (OVX). They transfected female rat bone marrow mesenchymal stem cells (BMMSCs) with miR-30a-3p mimics or inhibitors in the experiment. In a study, miR-30a-3p was found to target SFRP1. A negative correlation was found between miR-30a-3p and SFRP1 after ovariectomy. In the OVX group, the micro-CT results showed a significant reduction in bone mineral density (BMD). In addition, miR-30a-3p inhibitors promoted osteogenic differentiation in vitro and bone formation in vivo.

### 4.2. miRNAs Related to Promoting Osteogenesis

#### 4.2.1. miR-1-3p

It was found that miR-1-3p has a different effect on osteoporosis patients, as its level was significantly reduced. The direct target gene of miR-1-3p is SFRP1 [60]. As mesenchymal stem cells (MSCs) differentiate into osteogenic and adipogenic cells, miR-1-3p plays a crucial role in this process. A miR-1-3p overexpression stimulates osteogenesis and inhibits adipogenesis in murine mesenchymal stem cells (mMSCs), while a miR-1-3p suppression increases osteoclast activity and decreases bone mass [60].

#### 4.2.2. miR-210

Ren et al. investigated the effects of miR-210 on osteoporosis in postmenopausal rats using an OVX rat model and found that it was downregulated in OVX rats, and its increased expression alleviated osteoporosis. MiR-210 may modulate VEGF/Notch1 signaling activity, according to studies [61].

#### 4.2.3. miR-31

In reports by Mizoguchi et al., miR-31 is identified as an important miRNA in osteoclast regulation. As they discovered, miR-31 inhibits osteoclastogenesis via both excessive and insufficient activity of RhoA. MiR-31, whose expression was stimulated by RANKL with a RhoA target, controlled the processes of osteoclastogenesis and bone resorption by affecting the organization of the cytoskeleton [68].

### 4.3. miRNAs Related to Osteoclast Differentiation

**Figure 3:**
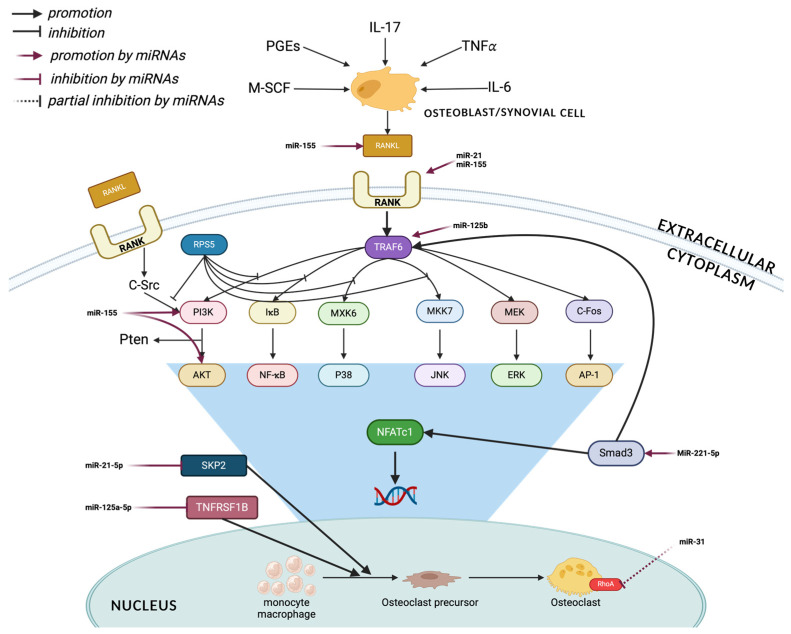
Schematic diagram illustrating the molecular mechanisms underlying osteoclast-associated microRNAs that target intracellular and extracellular targets in osteoporosis. Created with BioRender.com.

As adults, osteoclasts play a key role in skeletal problems and bone remodeling through bone resorption. Their ability to reabsorb bone tissue distinguishes them from other types of cells [82]. Regulation of osteoclasts is known to be mediated by several miRNAs [83,84]—Figure 3. 

#### 4.3.1. miR-125a-5p

A study on RAW 264.7 cells showed miR-125a-5p inhibited proliferation, migration, and invasion. High levels of miR-125a-5p in osteoclasts inhibit differentiation, likely by suppressing TNFRSF1B expression. MiR-125a-5p upregulation inhibited TNFRSF1B protein expression and promoted osteoclast differentiation, whereas miR-125a-5p downregulation did the opposite [62].

#### 4.3.2. miR-21-5p

Huang et al. used gene chip technology to screen for miRNAs differentially expressed during osteoclast differentiation. Their findings suggest that miR-21-5p plays a role in osteoclastogenesis by significantly reducing its levels in mature osteoclasts. A significant molecular inhibition of osteoclast differentiation and activity was demonstrated by in vitro experiments with miR-21-5p overexpression. A predicted target gene of miR-21-5p, SKP2, was significantly upregulated during osteoclast differentiation, and its overexpression reversed miR-21-5p’s inhibitory effect [63].

#### 4.3.3. miR-155

As reported by Mao et al., osteoporosis is associated with high miR-155 levels. The experiment examined the expression of MiR-155 and several other factors, including LEPR, AMPK, p-AMPK, RANKL, OPG, M-CSF, RANK, TRAP, Bax, Bcl-2, TNF-α, and IL-1β. In osteoporotic mouse models, miR-155 overexpression was associated with reduced bone density and tension. A miR-155 target, LEPR, was also found. In osteoporotic mice treated with alendronate, miR-155, which is upregulated via LEPR via AMPK activation, was reduced, and osteoclast activity and bone resorption were reduced [65]. Bone metabolism may be modulated by the autophagy pathway, which may affect skeletal structure. MicroRNAs (miRs) are important regulators of autophagy in recent studies. As a result, the hypothesis was developed that inflammation could induce miR-155 expression, which in turn induced autophagy in osteoclasts (OCs), causing bone loss.

A study by Sul et al. found that miR-155 expression was increased in mice with LPS-injected tibiae and in OCs stimulated by lipopolysaccharide (LPS). By binding to the 3′-UTR region of TAB2, miR-155 interacts with BECLIN1, an important target gene for transforming growth factor-associated protein 1 [64]. This results in BECLIN1 dissociating from TAB2, which then causes TAB2 to associate with TAK1 during autophagy induction. As a result, LPS-induced miR-155 stimulated autophagy by reducing TAB2 expression. Based on the study findings, miR-155 might play an important role in regulating LPS-induced autophagy in OC by interacting with TAB2. The inhibition of miR-155 expression may therefore be a potential therapeutic target to limit OC differentiation in inflammatory conditions [64].

#### 4.3.4. miR-182

In a study of inflammatory osteoclastogenesis, miR-182, a novel microRNA derived from mouse bone marrow (BMM)/osteoclast precursors, played an important role. It has been shown that MiR-182 promotes osteoclastogenesis induced by TNF-α. As well, miR-182 expression is regulated by RBP-J. Its reduction leads to a significant inhibition of the enhanced osteoclastogenesis program induced by TNF-α in RBP-J-deficient cells [26]. In follow-up studies, miR-182 was found to positively regulate the osteoclastogenic transcription factors NFATc1 and Blimp1. Furthermore, miR-182 direct targets, Foxo3 and Maml1, play a significant inhibitory role in osteoclastogenesis under the influence of TNF-α. Therefore, regulation of miR-182 by RBP-J is a key mechanism that promotes TNF-α-induced osteoclastogenesis through inhibition of Foxo3 and Maml1 [26].

A molecular mechanism by which miR-182 regulates osteoblast proliferation and differentiation was analyzed by Kim et al. This was accomplished using the following programs: Target scan, Pictar, and miRBase. Observations led them to select FoxO1 as a potential miR-182 target gene. As a negative regulator of osteogenesis, MiR-182 suppresses FoxO1 expression. As a result, osteoblasts are not able to multiply and differentiate [66].

#### 4.3.5. miR-221-5p

Based on serum from osteoporosis patients, Guo et al. found that postmenopausal osteoporosis patients had decreased miR-221-5p expression compared to a healthy control group. During osteoclastogenesis, miR-221-5p expression was downregulated, and Smad3 expression was upregulated. Inhibition of osteoclastogenesis by miR-221-5p and promotion by Smad3 were observed. The results of this study suggest that miR-221-5p is effective in alleviating postmenopausal osteoporosis by suppressing osteoclastogenesis via Smad3, providing new ideas for molecular-targeted osteoporosis treatment [67].

### 4.4. miRNAs Related to the Inhibition of Osteogenesis

#### 4.4.1. miR-133a

Using bioinformatics analysis, Wang et al. identified three potential miR-133a target genes that inhibit osteoclastogenesis—CXCL11, CXCR3, and SLC39A1. There was a negative correlation between all three genes and miR-133a, though it was not statistically significant. Nevertheless, these genes were consistently upregulated in both high and low bone mineral density (BMD) groups [69]. MiRNAs normally regulate the expression of hundreds of genes, making each gene’s regulatory effect relatively small. It is important to note, however, that osteoporosis is a complex disease regulated by many genes.

#### 4.4.2. miR-25-3p

In an experiment using bone marrow mesenchymal stem cells (BMSC) in patients with osteoporosis (OP), the outflow of the miR-25–3p/ITGB3 axis was examined. There was a significant increase in miR-25-3p levels in osteoporosis patients compared with healthy patients. It has also been shown that miR-25-3p targets ITGB3, which leads to the inhibition of osteogenic differentiation of bone marrow stem cells in patients with osteoporosis [70]. Also of interest is the report by Ni et al., which indicates that miR-25-3p is strongly upregulated in T2DM-induced osteoporosis. Moreover, this study confirms saliva provides a non-invasive method for detecting miRs in osteoporosis [85].

#### 4.4.3. miR-125b

In the study by Wang et al., miR-125b levels were significantly higher in BMSCs from osteoporotic rats. BMSCs transfected with miR-125b mimic were found to have reduced osteogenesis differentiation in the in vitro assay. Moreover, agomiR-125 resulted in more severe osteoporosis in postmenopausal rats with osteoporotic bone tissue morphology and serum ALP and OC levels based on in vivo results. According to bioinformatic predictions and the tests used, miR-125b is directly targeting Smad4 [71].

According to Wang et al., miR-125b is overexpressed in postmenopausal osteoporosis based on microarray analysis of miRNA profiles collected from postmenopausal women’s tissue samples. Researchers have demonstrated that miR-125b inhibits cell viability, promotes the release of LDH, increases the ratio of RANKL to OPG, and inhibits BMP2 and Runx2. TRAF6 has been identified as a potential target of miR-125b via the JAK2/STAT3 pathway by bioinformatics [72].

#### 4.4.4. miR-146a

For TNF-α applications in bone marrow mesenchymal stem cells (BMSCs), Kuang et al. observed a significant increase in miR-146 expression. As a result of increased miR-146a expression, osteogenesis was inhibited. Posttranscriptionally, MiR-146a downregulates Smad4, an essential mediator of the BMP pathway [73]. These findings may help explain why osteoporosis is aggravated by inflammation. 

### 4.5. miRNAs Associated with Other Mechanisms of Bone Loss

#### 4.5.1. miR-21

Another microRNA under investigation is miR-21. Studies in the female population showed that miR-21 concentration was significantly higher in the serum of postmenopausal women with osteoporosis compared to women without osteoporosis. A negative effect of miR-21 expression in serum on bone mineral density (BMD) values of the spine in postmenopausal women with osteoporosis was also demonstrated [74]. There seems to be a link between low estrogen levels in postmenopausal women and an increase in miR-21 expression in serum. High miR-21 expression increases RANKL production and reduces OPG and TGF-β1, which ultimately increases bone resorption and reduces BMD, causing osteoporosis. Interestingly, this study also showed that moderate physical activity inhibited serum miR-21 expression [74].

#### 4.5.2. miR-195

Based on the results of Gu et al., miR-195 could target and regulate GIT1 expression in human chondrocyte cells (CHON-002). This study found that miR-195 inhibited chondrocyte cell proliferation and migration, possibly by regulating the expression of GIT1 [75].

## 5. MicroRNA Clinical Application for Osteoporosis

Due to the asymptomatic course of osteoporosis, it is diagnosed in patients already at an advanced stage of the disease, which may lead to serious consequences such as impaired functioning and disability. Therefore, the development of an effective diagnostic approach that allows detection of the disease before its advanced stage and a new therapeutic approach development is important [5]. In this section, we provide a review of promising perspectives and the current status of the application of microRNA in osteoporosis treatment and diagnosis.

The approach using microRNA and targeting specific processes involved in the pathogenesis of osteoarthritis seems to be a promising therapeutic approach for use in therapy [86,87].

MicroRNA-based therapy may be targeted for suppressing the activity of overexpressed miRNA (loss-of-function) or for restoring the expression of down-regulated or non-functional miRNA (gain-of-function), whose activity is disturbed as a result of the pathogenesis of osteoporosis. To block the activity of specific miRNA, miRNA sponges, miRNA masks, and anti-miRNA oligonucleotides (anti-miRs), while restoring specific miRNA—miRNA mimics and viral vectors are being considered [86,87]. However, selecting accurate miRNA targets is crucial, and understanding the dynamics and involvement of miRNA in the pathogenesis of osteoporosis is also crucial. In Table 3, we present collected data on miRNA analysis from patient samples with osteoporosis that are suggested for potential use in miRNA-based diagnostics and therapy published in the last four years because there are currently well-written review papers collecting data from before 2021 [88,89,90].

MicroRNA can be used as an early diagnostic biomarker for osteoporosis but also for monitoring the progression of this disease [88]. Currently, one miRNA panel proposed as a candidate for the diagnostic tool for osteoporosis is commercially available. OsteomiR is specific to 19 bone-related miRNAs, which allow for the evaluation of the fracture-risk index of osteoporotic patients [87]. It has been shown that this panel has high accuracy and can be used as a diagnostic tool for osteoporosis. Moreover, using this panel, Kerschan-Schindl et al. selected two biomarkers from patients’ serum—miR-375 as an indicator of high risk of osteoporosis for women after menopause and miR-203a as a diagnostic marker for fragility fractures [91].

It is worth noting here that currently used pharmacological agents in the treatment of osteoporosis can affect miRNA expression. Li et al. showed that long-term use of bisphosphonates by patients with osteoporosis can suppress bone formation and inhibit osteoblastic formation via overexpression of miRNA-30a-5p. Therefore, it can be a biomarker for monitoring osteogenesis treatment as well as be a potential target for therapy [92]. Similarly, Messner et al. analyzed miRNA from patients’ serum with osteoporosis treated with Denosumab. They selected three (miR-454-3p, miR-26b-5p, and miR-584-5p), which were defined by the authors as top biomarker candidates for osteoporosis [93].

In 2022, Zhao et al., in turn, used the miRNA microarray technique to evaluate that miR-144-5p, miR-506-3p, miR-8068, and miR-6851-3p may be a biomarker for postmenopausal osteoporosis, but only miR-144-5p may be a predictive biomarker for changes in bone mineral density in lumbar spine 1–4, total hip, and femoral neck. They also performed bioinformatic analysis, suggesting that expressed miRNAs were targeted to genes such as *YY1*, *VIM*, and *YWHAE* strongly involved in bone metabolism processes. Importantly, the authors deposited microarray data in the Gene Expression Omnibus (GEO) database [21]. In turn, in 2023, Al-Rawaf et al. showed that miRNA correlated with osteocalcin, bone-specific alkaline phosphatase, and deoxypyridinoline such as miR-21, miR-24, and mir-100 were upregulated, while miR-24a, miR-103-3p, and miR-142-3p were downregulated [94]. Similarly, Mohammadisima et al. showed upregulation of miR-21-5p but also miRNA-422a in osteoporotic plasma samples [95].

Recently, Sun et al. investigated the exosomal miRNA potential for osteoporosis diagnosis using exosomal fractions from plasma samples from 26 patients with osteoporosis and 21 non-osteoporotic volunteers. Results showed that a combination of three miRNAs, such as miR-34a-5p, miR-9-5p, and miR-98-5p, is characterized by high diagnostic performance; however, the authors suggest that a larger population should be tested to validate this discovery [96].

**Table 3:** Data on miRNA analysis from osteoporosis patient samples that may be useful for miRNA-based diagnostics and therapies (collected data published in years 2021–2024). MiRNAs, their expression, sample type and analysis method were included.

miRNA	Expression Changes	Sample Type	Method of Analysis	References
miRNA-30a-5p	Up	Serum	GeneChip miRNA array	[92]
miR-144-5p miR-506-3p miR-8068 miR-6851-3p	Up	Serum	miRNA microarray, Real-time-PCR	[21]
Exosomal: miR-34a-5p miR-9-5p miR-98-5p	Up	Plasma	Next-Generation Sequencing, Real-time-PCR	[96]
miR-375 miR-203a	N/A	Serum	osteomiR^®^ RUO assay	[91]
miR-21 miR-24 mir-100	Up	Serum	Real-time-PCR	[94]
miR-24a miR-103-3p miR-142-3p	Down	Serum	Real-time-PCR	[93,94]
miR-454-3p miR-26b-5p miR-584-5p	Up	Serum	Next-Generation Sequencing, qRT-PCR	[93]

The miRNA analysis connected with bone metabolism in patients with osteoarthritis is under evaluation by 11 registered clinical trials in the ClinicalTrials.gov database—Table 4. Some of those studies aim to analyze the involvement of classic treatment on the miRNA profile of patients with osteoporosis, or using different food supplements, while others mainly focus on different conditions such as type 1 diabetes or primary hyperparathyroidism with connection to osteoporosis, bone condition, and metabolism.

## 6. Conclusions

MiRNAs are currently the subject of much research regarding how they contribute to osteoporosis pathogenesis. The role of tissue miRNA in intracellular processes has been demonstrated in many studies. These processes include apoptosis, cell proliferation, cell migration, differentiation, metabolism, angiogenesis, oncogenesis, and cell cycle control [12,97,98]. There is increasing evidence that microRNAs (miRNAs) are important in the pathophysiology of some diseases, such as osteoporosis. There are a number of miRNAs that regulate bone metabolism, including osteoblast and osteoclast differentiation, bone matrix synthesis, and bone resorption. Some miRNAs are associated with osteoblast and osteoclast differentiation, balancing bone formation and resorption. For example, miR-185, miR-185-5p, miR-124, miR-451a, miR-133a, miR-33-5p, miR-25-3p, miR-21, miR-1-3p, miR-125a-5p, miR-146a, miR-195, miR-103a, miR-194, miR-182, miR-155, miR-29b, miR-199b-5p have been identified as key regulators of osteoblast and osteoclast differentiation. The dysregulation of these miRNAs may result in disturbances in bone metabolism, leading to osteoporosis. As an example, miR-29 regulates collagen expression, which plays an important role in bone formation. When these miRNAs are disrupted, osteoporosis-related conditions such as decreased bone mineral density and weakened bone structure can occur.

Furthermore, miRNAs may play an important role in the regulation of signaling pathways related to bone resorption. For instance, miR-155 can modulate osteoclastic activity by affecting the Receptor Activator of Nuclear Cells (RANKL) pathway. Factor κB Ligand)/RANK (Receptor Activator of Nuclear Factor κB/OPG (Osteoprotegerin). Thus, dysregulation of miRNAs that regulate the differentiation and activity of osteoblasts and osteoclasts, as well as signaling pathways linked to bone metabolism, may contribute to osteoporosis.

These mechanisms may be used to develop innovative therapeutic and diagnostic strategies for osteoporosis. Because of their high conservation, detectability, and specific spatiotemporal expression in serum, specific miRNAs may also prove useful as osteoporosis biomarkers. They are therefore promising candidates for liquid biopsy [99,100]. By using miRNAs as biomarkers, osteoporosis and fracture risk can be assessed and detected quickly and effectively. The advantage of miRs in the diagnosis of osteoporosis is that some miRs can be detected in patients’ saliva, which is a non-invasive test.

Despite this, miRNA-based drugs still suffer from one important limitation. In reality, miRNAs do not follow a one-to-one correspondence; rather, their effects are multiplicative. Therefore, a particular miRNA may target many different genes, causing potential side effects [63].

The identification of new therapeutic targets and the development of more effective treatments for osteoporosis may be possible through understanding the mechanisms governing miRNA regulation. Hence, it is extremely important to continue to investigate characteristic miRNAs in osteoporosis patients. It has also been suggested that modulating certain miRNAs might be a potential therapeutic strategy for osteoporosis. The modulation of miRNAs may affect bone matrix synthesis, bone resorption, and osteoblast differentiation and differentiation.

Further development of osteoporosis diagnostics and treatment depends on further clinical studies on the presence of characteristic miRNAs and their impact on individual genes/signal pathways, as well as research on miRNA modulation as therapeutic targets. Using miRNAs as diagnostic and therapeutic tools may improve osteoporosis management and reduce its negative outcomes.

## Figures and Tables

**Table 1:** The risk factors contributing to osteoporosis development.

Group of Factors	Risk Factors		References
Demographic	Female; Caucasian and Asian		[34]
Genetic	Slim body structure and low body weight determined by genetics; family predispositions, such as parents’ history of hip fractures		[34]
Reproductive status	Postmenopausal period; Nullipara; Late menarche; Early menopause; Hypogonadism; Various etiologies of sex hormone deficiency		[34,35]
Diet and lifestyle	Low calcium supply; Insufficient or excessive phosphorus supply; Protein deficiencies; Diets that contain too much protein; Reduced supply of vitamin D;		[34,35]
Body weight	Body mass index (BMI) < 20		[35]
Physical activity	A lifestyle of reduced physical activity; Immobilization		[34]
Environmental	No sunlight exposure; Smoking: active and passive; Alcohol abuse; Excessive caffeine consumption;		[34,35]
Medicines	Glucocorticoids administered for 3 months (>7.5 mg prednisolone equivalent); T3 and T4 hormones; Anticonvulsants; Stomach protection products containing aluminum; Proton pump inhibitors (PPIs); Vitamin K antagonists; Heparin; Tetracyclines; Isoniazid; Immunosuppressive drugs; Anticancer drugs including aromatase inhibitors		[34,35]
Diseases	Endocrine and metabolic	Hyperparathyroidism; Hyperthyroidism; Obesity and type II diabetes; Cushing’s syndrome; Hyperprolactinemia;	[34,35,36]
	Digestive tract	Celiac disease and other malabsorption disorders; Inflammatory bowel diseases: Crohn’s disease and Ulcerative Colitis, Pancreatic diseases;	
	Kidneys	Renal osteopathy;	
	Hematological	Monoclonal gammopathies: Gammopathy of unclear significance [MGUS], multiple myeloma [MM]; Mastocytosis;	
	Rheumatological	Rheumatoid arthritis (RA)	
	Congenital connective tissue diseases	Ehlers-Danlos; Marfan syndrome;	
	Genetic syndromes	Cystic Fibrosis; Hemochromatosis; Hypophosphatasia; Menkes syndrome; Porphyria; Gaucher’s disease; Hypophosphatemia; Homocystinuria	

**Table 2:** The role of selected microRNAs in bone turnover—miRNA expression in the experiment, the experimental model, effect, potential target genes/pathways, and a possible attitude towards osteoporosis.

microRNA	miRNA Expression in the Experiment	Experimental Model	Action	Potential Target	A Possible Attitude toward Osteoporosis	References
miRNAs related to osteoblast differentiation and proliferation						
miR-185	downregulated—in mice miR-185-knockout, upregulated—in mice with osteoporosis after bilateral ovariectomy (OVX)	animal models: mouses miR-185-knockout (KO); mouse model OVX	inhibition of osteoblast growth and proliferation—OVX mouse model; increasing the viability of osteoblasts and reducing cell apoptosis—KO mouse model	BMP/Smad biglycan (BGN), axis Wnt /β-catenin, PTN gene	upregulated	[41]
miR-124	upregulated	human cell line (BMSC) mouse cell line (BMSC and C2C12)	inhibition of differentiation osteoblasts	Dlx3, Dlx5, Dlx2	upregulated	[42]
	upregulated	animal model—mice (ICR)	inhibition of osteoclast precursor proliferation and migration	NFATc1, RhoA i Rac1	downregulated	[43]
	upregulated	human cells	promoting the differentiation of fibroblasts into osteoblasts	GSK-3β, Wnt/β-catenin pathway	N/A	[44]
miR-33-5p	upregulated	animal model—mouses (MC3T3-E1)	promoting osteoblast differentiation	Hmga2	downregulated	[45]
miR-103a	upregulated	human cell line (hFOB)	inhibition of osteoblast differentiation and their activity, inhibition of matrix mineralization	Runx2	upregulated	[46]
miR-139-5p	upregulated	human cell line (hBMSCs)	inhibition of osteogenic differentiation BMSCs	CTNNB1, FZD4	upregulated	[47]
miR-194	upregulated	animal model—mice (BMSC)	promoting osteoblast differentiation	STAT1, Runx2	downregulated	[48]
miR-199b-5p	upregulated	human cell line (BMSC)	positive regulation of osteoblast differentiation of BMSCs	ALP, Runx2, GSK-3β/β-catenin signaling pathway	downregulated	[49]
	upregulated	human cells (hSCAP)	promoting osteogenesis	IFIT2	downregulated	[50]
miR-185-5p	upregulated	mouse cells ameloblast-like (LS8) and the osteoblastic cell line (MC3T3-E1)	inhibition of differentiation of osteoblasts and ameloblasts	Dlx2	upregulated	[51]
miR-542-3p	downregulated	animal model—rat with ovaries (OVX)	inhibition of osteoblast formation and differentiation	SFRP1	downregulated	[52]
miR-146a	downregulated	animal model—female rats	promoting osteoblast formation	Wnt/β-catenin	downregulated	[53]
miR-128	upregulated	human femoral neck tissue, mouse cell line (C2C12)	inhibition of osteoblast differentiation	SIRT6	upregulated	[54]
miR-183	upregulated	animal model—mice with ovaries (OVX)	inhibition of osteoblast differentiation	Smad4	upregulated	[55]
miR-99b-5p	upregulated	human tissue from the neck of the femur	inhibition of osteoblast proliferation and differentiation	FGFR3	upregulated	[56]
miR-137	upregulated	animal model—rats	lowering the level of osteoblasts and reducing bone mineral density	RUNX2	upregulated	[57]
miR-29b	upregulated	mouse osteoblast model	promoting osteoblast differentiation, suppressing collagen synthesis in mature osteoblasts to maintain a differentiated phenotype	COL1A1, BMP1 and ADAM12	downregulated	[58]
miR-30a-3p	upregulated	animal model—rat with ovaries (OVX)	decreased bone mass and bone formation and increased bone loss in OVX rats	SFRP1	upregulated	[59]
miR-451a	upregulated	animal model—miR-451a-knockout (KO) mice, mouse model WT, mouse model OVX	weakening of the bone structure, lowering the level of collagen	Bmp6/SMAD1/5/8	upregulated	[16]
miRNAs related to promoting osteogenesis						
miR-1-3p	up-regulated	human tissue	promoting osteogenesis and adipogenesis, regulating bone formation	SFRP1	downregulated	[60]
miR-210	up-regulated	animal model—rat with ovaries (OVX)	promoting osteogenic differentiation of BMSCs, improving the microstructure of bone tissue, regulating bone formation and resorption, promoting osteoblast differentiation	VEGF/Notch1	downregulated	[61]
miRNAs related to osteoclast differentiation						
miR-125a-5p	up-regulated	mouse macrophage cell line (RAW 264.7) and human cell line (293T)	promoting osteoclast differentiation	TNFRSF1B	upregulated	[62]
miR-21-5p	up-regulated	mouse macrophage cell line RAW264.7	inhibition of osteoclast differentiation and activity	SKP2	downregulated	[63]
miR-155	up-regulated	animal model—mouses (C57BL/6J)	enhancing autophagy, differentiation, and osteoclast activity	TAB2	upregulated	[64]
	up-regulated	animal model—mice with osteoporosis	promote osteoclast differentiation	LEPR gene, OPG/RANK/RANKL, AMPK	upregulated	[65]
miR-182	up-regulated	animal model—mouses	promote osteoclast differentiation	Foxo3, Maml1	upregulated	[26]
	down-regulated	mouse cell lines (C3H10T1/2, MC3T3-E1)	reducing cell viability, increasing cell apoptosis, inhibiting osteoblast differentiation, inhibiting osteogenesis	FoxO1, PI3K/AKT	upregulated	[66]
miR-221-5p	downregulation—serum of patients with osteoporosis, upregulation—macrophages treated with miR-221-5p mica	human serum, macrophages RAW264.7	inhibition of osteoclastogenesis	Smad3	downregulated	[67]
miR-31	downregulated	animal model—male mice	suppression of osteoclast function, inhibition of osteoclast differentiation, negative regulation of osteoclast cytoskeleton organization	RhoA	N/A	[68]
miRNAs related to the inhibition of osteogenesis						
miR-133a	upregulated	human tissue	inhibition of osteogenesis	N/A	upregulated	[69]
miR-25-3p	upregulated	human bone marrow cells	inhibition of osteogenic differentiation of BMSCs	ITGB3	upregulated	[70]
miR-125b	upregulated	rat cell line (BMSC)	inhibition of osteogenic differentiation of BMSCs	Smad4	upregulated	[71]
	upregulated	human tissue	increasing RANKL expression and osteoclast differentiation	TRAF6	upregulated	[72]
miR-146a	upregulated	animal model—mouses	inhibition of osteogenic differentiation	Smad4	upregulated	[73]
miRNAs associated with other mechanisms of bone loss						
miR-21	upregulated	human tissue	promoting bone resorption	RANKL, TGF-β 1, OPG	upregulated	[74]
miR-195	upregulated	human chondrocyte cell line (CHON-002)	inhibition of chondrocyte proliferation and migration	GIT1	upregulated	[75]

**Table 4:** Clinical trials of miRNAs related to osteoporosis patients. An overview of the clinical trials is pro-vided, including the number, title, status, and description (collected data published in years 2021–2024).

NCT no.	Study Title	Status	Purpose/Aim	Results
NCT05556499	The Bone-parathyroid Crosstalk in Primary Hyperparathyroidism (PARABONE)	Not yet recruiting	Investigation of the interaction between bone and parathyroid glands in patients with primary hyperparathyroidism with analysis of circulating lncRNAs and miRNAs in relation to the osteo-metabolic state.	N/A
NCT05912309	Effects of Time-restricted Eating and Exercise Training on Skeletal Muscle Mass Quantity, Quality, and Function in Postmenopausal Women With Overweight and Obesity	Recruiting	Investigation of the effect of restricted eating and exercise on skeletal muscle tissue quantity, resting energy expenditure, and cardiometabolic health as well as miRNA biomarkers analysis in postmenopausal women with obesity, menopause-related conditions, sarcopenia, and osteoporosis.	N/A
NCT05328154	MAGnesium Effect With ANtiosteoporotic Drugs (MAGELLAN)	Recruiting	Demonstration of the superiority of the combination of bisphosphonates and magnesium over bisphosphonates alone in postmenopausal osteoporosis with analysis of epigenetic biomarkers for osteoporosis in women.	N/A
NCT05228262	Vascular Function, Sarcopenia, and Pain in Postmenopausal Osteoporosis (VASCO)	Recruiting	Evaluating the impact of antiosteoporosis drugs on the osteoporosis-cardiovascular-sarcopenia triad and on pain with the secondary aim of studying the epigenetic biomarkers in osteoporosis in women.	N/A
NCT02705040	Roles of microRNAs in the Development of Osteoporosis in Men—Preliminary Study	Unknown status	Observational, prospective study evaluating the role of specific miRNA in men with osteoporosis.	N/A
NCT05673837	the Type ONe dIabetic Bone Collaboration Study	Active, not recruiting	Examination of bone properties of patients with Type 1 diabetes with the analysis of miRNA correlated with bone metabolism. Investigating epidemiology of osteoporosis in Type 1 Diabetes patients.	N/A
NCT03931109	Circulating microRNA Signatures in Primary Hyperparathyroidism	Active, not recruiting	Prospective, non-randomized pilot study on expression levels of miRNA in patients’ serum with primary hyperparathyroidism with and without osteoporosis.	N/A
NCT02128009	Study on the microRNA Expression Level in Postmenopausal Osteoporosis (microRNA)	Completed	Investigating molecular mechanism by analysis of miRNA levels in postmenopausal osteoporosis with kidney yin deficiency syndrome.	N/A
NCT03472846	MiDeTe—microRNA Levels Under Denosumab and Teriparatide Therapy in Postmenopausal Osteoporosis	Completed	Detection of bone-specific miRNAs concentration in serum of 26 women with postmenopausal osteoporosis under antiresorptive or osteoanabolic treatment.	N/A
NCT01875458	Biomarker Identification in Orthopedic and Oral Maxillofacial Subjects	Completed	Identification of DNA and miRNA biomarkers which may play a role in differential metabolism in response to drugs with Bisphosphonate.	N/A
NCT05421819	Design and Development of a Novel Food Supplement for Osteoporosis Based on Gut Microbiome Mechanisms (OSTEOME)	Completed	Investigation of the effect of novel food supplement based on gut microbiome mechanism in patients with osteoporosis (with analysis of miRNAs in patients’ serum).	N/A

N/A—not applicable.

## Abbreviations

miRNA, mRNA, miR	microRNA
OPG	osteoprotegerin
BMD	bone mineral density
BMSC	bone marrow mesenchymal stem cells
CMS	cyclic mechanical stretching
CTNNB1	catenin beta 1 gene
FoxO1	forkhead box protein O
FZD4	frizzled Class Receptor 4
MSC	mesenchymal stem cells
N/A	not applicable
NFATc1	nuclear factor of activated T cells 1
mMSC	mouse mesenchymal stem cells
Rac1	Rac family small GTPase 1
RANK	receptor, activator of the ligand of nuclear factor κB
RANKL	ligand of the receptor that activates nuclear factor NF-κB
RhoA	Ras homolog family member A
TNFRSF1B	gene 1B, a member of the TNF receptor superfamily
Smad4	mothers against decapentaplegic homolog 4
TNF-α	tumor necrosis factor α
M-CSF	macrophage colony-stimulating factor
TRAP	tartaric acid phosphatase
LEPR	leptin receptor
AMPK	adenosine monophosphate-activated protein kinase
p-AMPK	phosphorylated activated adenosine multiphosphate protein conase
OVX	ovariectomized
OP	osteoporosis
SIRT6	silencing information regulatory protein 6
TAB 2	TAK1-binding protein 2
IFIT2	interferon-induced protein with Tetratricopeptide Repeats 2
ALP	alkaline phosphatase
